# Examining assumptions regarding valid electronic monitoring of medication therapy: development of a validation framework and its application on a European sample of kidney transplant patients

**DOI:** 10.1186/1471-2288-8-5

**Published:** 2008-02-19

**Authors:** Kris Denhaerynck, Petra Schäfer-Keller, James Young, Jürg Steiger, Andreas Bock, Sabina De Geest

**Affiliations:** 1 Institute of Nursing Science, University of Basel, Bernoullistrasse 28, 4056, Basel, Switzerland; 2 Division of Transplant Immunology and Nephrology, University Hospital, Petersgraben 4, 4031 Basel, Switzerland; 3 Basel Institute for Clinical Epidemiology, University Hospital, Hebelstrasse. 10, 4031 Basel, Switzerland; 4 Division of Nephrology, Cantonal Hospital, Buchserstrasse, 5000 Aarau, Switzerland

## Abstract

**Background:**

Electronic monitoring (EM) is used increasingly to measure medication non-adherence. Unbiased EM assessment requires fulfillment of assumptions. The purpose of this study was to determine assumptions needed for internal and external validity of EM measurement. To test internal validity, we examined if (1) EM equipment functioned correctly, (2) if all EM bottle openings corresponded to actual drug intake, and (3) if EM did not influence a patient's normal adherence behavior. To assess external validity, we examined if there were indications that using EM affected the sample representativeness.

**Methods:**

We used data from the Supporting Medication Adherence in Renal Transplantation (SMART) study, which included 250 adult renal transplant patients whose adherence to immunosuppressive drugs was measured during 3 months with the Medication Event Monitoring System (MEMS). Internal validity was determined by assessing the prevalence of nonfunctioning EM systems, the prevalence of patient-reported discrepancies between cap openings and actual intakes (using contemporaneous notes and interview at the end of the study), and by exploring whether adherence was initially uncharacteristically high and decreased over time (an indication of a possible EM intervention effect). Sample representativeness was examined by screening for differences between participants and non-participants or drop outs on non-adherence.

**Results:**

Our analysis revealed that some assumptions were not fulfilled: 1) one cap malfunctioned (0.4%), 2) self-reported mismatches between bottle openings and actual drug intake occurred in 62% of the patients (n = 155), and 3) adherence decreased over the first 5 weeks of the monitoring, indicating that EM had a waning intervention effect.

**Conclusion:**

The validity assumptions presented in this article should be checked in future studies using EM as a measure of medication non-adherence.

## Background

**Figure 1:**
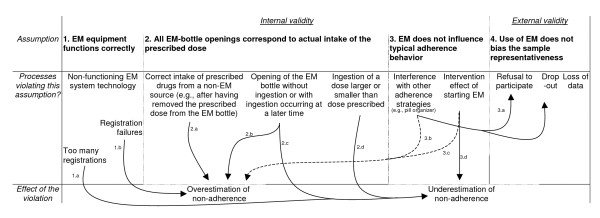
Overview of possible violations of the assumptions underlying internal and external validity of EM: effects on the non-adherence estimate.

The introduction of electronic monitoring (EM) for assessing medication non-adherence has enabled researchers and clinicians to gather detailed data about medication-taking behavior. EM systems use pill bottles containing a small electronic processor that records the date and time of each cap opening, resulting in a more detailed non-adherence measurement. Compared to other methods (e.g., assay, self-report, collateral report, prescription refills), EM captures more of the dynamics of medication-taking behavior [1]. Although EM has for this reason been used as gold-standard method for assessing medication adherence [2,3], empirical evidence and clinical experience suggest that several factors can jeopardize the internal and external validity of EM studies [4]. Unbiased EM measurement depends on the fulfillment of at least four assumptions. The first 3 of these assumptions ensure internal validity: 1) correct functioning of the EM equipment, 2) correspondence between EM-bottle openings and actual intake of the prescribed dose, 3) and absence of an EM-associated influence on a patient's normal adherence behavior. The fourth assumption ensures external validity: use of EM does not bias the representativeness of the sample. This article discusses processes that might lead to a violation of these assumptions and describes how the assumptions were empirically tested. Figure 1 outlines the possible effects of these assumption-violating processes on adherence measurement (i.e., whether they lead to overestimation or underestimation of non-adherence).

### Assumptions underlying valid electronic medication adherence monitoring: Internal validity

#### Assumption 1

The first assumption ensuring unbiased assessment of medication non-adherence requires that electronic monitoring equipment function properly (Figure 1). Quality tests of the widely used Medication Event Monitoring System (MEMS^®^-6, Aardex Ltd.) revealed that the system performed well under normal or extreme laboratory conditions (i.e., if exposed to heat, cold, shocks, or water). A failure rate of below 0.5% is reported [5]. Reports of how MEMS performs in the field show a similar pattern. A two-month assessment of eleven purposively sampled MEMS-IV bottles used in a one-year study of HIV patients (in which a number of bottles were suspected of being damaged) showed that EM failed to register only 2.5% of the generated events [6]. Non-functional MEMS-V caps are also noted in an EM study in kidney transplant patients [7]. The exact number of non-functional caps, however, could not be inferred from these research reports.

#### Assumption 2

The second assumption ensuring unbiased assessment of medication non-adherence requires that each time the patient unscrews the EM-bottle cap he/she also ingests the prescribed dose immediately. Validity of EM data can be affected because of discrepancies in pill removal and actual ingestion time (Figure 1). If patients correctly ingest the immunosuppressive medication either from a source other than the EM bottle or from a supply of pills previously removed from the EM bottle [6,8], for example because the EM bottle is unpractical or embarrassing for privacy reasons [9,10], non-adherence will be overestimated. This also includes patients who "trigger" their bottles while taking medications from another source, but who remove and replace the cap too quickly for an opening to be recorded (see Figure 1, arrow 2.a). Under- or overestimation may also occur when patients open the EM bottle but do not remove any pills, as has been reported in 26% of patients on HIV medication (e.g., to demonstrates the EM system to friends; Figure 1, arrow 2.b/c) [11,6]. Likewise, ingesting doses that are larger or smaller than those prescribed will result in underestimation of non-adherence (Figure 1, arrow 2d).

Under- or overestimation of non-adherence can, to some extent, be prevented by asking patients to report discrepancies between cap openings and pill intakes and using these reports to adjust the raw EM data, or censor periods where the EM bottle was not used properly [8]. Several studies have implemented this method by giving patients a form on which to write down occurred discrepancies [9,12]. Drawbacks of this method is that it introduces bias itself [8]. Patients who are non-adherent to the medication therapy are also likely to keep poor records of discrepancies [13]. Also, asking patients to keep notes might induce self-monitoring and thus become an adherence-enhancing intervention.

#### Assumption 3

The third assumption underlying valid EM measurement requires the absence of an EM-induced effect on a patient's normal or typical adherence behavior (Figure 1). Two pathways are important in this regard. Firstly, EM may influence normal intake behavior because patients cannot use medication aids like pill organizers as usual and, at the same time, be electronically monitored [4]. Secondly, the awareness of being monitored may change the patient's typical adherence habits [14]. Using a pill organizer can increase the burden of a patient participating in an EM study, and lead to a lower participation to EM-studies among the pill-organizer users [4], or to stopping to use the pill organizer when continuing to be part of the study [15] (Figure 1, arrow 3.a). The problems related to combining an EM bottle and a pill organizer are expected to result in overestimating non-adherence (arrow 3.b). Support for the hypothesis that the awareness of being monitored may change a patient's typical medication-taking habits comes from patient reports indicating that being electronically monitored influences normal intake behavior. In most cases, patients reported an increased adherence, seldom a decreased one [16,9,12,18,4] (Figure 1, arrows 3.d & 3.c). Support from sources other than these patient reports is scarce. These sources are summarized in Table 1. Five intervention studies examined whether administering EM [14,16,19] or disclosing the monitoring purpose of EM [13,20] changed a patient's typical adherence behavior and one observational study examined whether non-adherence increased over time after having started EM [21]. The results from these studies were inconclusive. The studies of Elixhauser et al. (1990) and Bertholet et al. (2000) confirmed that starting EM alters adherence behavior, while the other studies could not find any difference. The latter finding most probably reflected the existence of methodological weaknesses, rather than the absence of an EM-related intervention effect. A study overcoming most of the methodological flaws of currently published studies should include a large enough sample, evaluate a possible intervention effect at different time points, use statistical tests properly, and adopt a non-adherence measurement method independent of a patient's awareness (thus not self-report).

**Table 1:** Published studies testing a possible intervention effect of electronic-medication monitoring on typical medication-taking behavior

**Author & publication year**	**Study design**	**Description of the sample**	**n**	**EM**	**Description of the intervention**	**Outcome variable: medication adherence or clinical outcome**	**Result**
Wagner et al. 2002	Randomized controlled trial	A community convenience sample of adult HIV-positive patients on HAART	117	MEMS	Experimental group received EM; control group did not	Adherence measured with self-report 4 weeks after study start (% of prescribed pills taken)	Less adherence in the EM group (91%) than in the control group (94%; p=.73)
	Pre-post intervention study	A community convenience sample of adult HIV-positive patients on HAART monitored with EM	60	MEMS	EM started after baseline blood pressure measurement	Adherence measured with self-report at baseline and after 4 weeks	Less adherence after introducing EM (91%) compared to baseline (93%; p=.16)
Bertholet et al. 2000	Pre-post intervention study	A convenience sample of primary care/hypertensive clinic patients with therapy-resistant hypertension	69	MEMS	EM started after baseline blood pressure measurement	Clinical outcome: blood pressure evaluation after 1 – 2 months	Blood pressure was lower after EM (14/9 cm Hg) compared to baseline (16/10 cm Hg; p < .001)
Matsui et al. 1994	Pre-post intervention study	A convenience sample of young β-thalassemia outpatients on a new iron chelator	10	MEMS	The purpose of EM was disclosed to patients after ± 11 months	Adherence measured by EM using the taking adherence parameter ± 18 months after disclosure	Greater adherence after disclosure (84%) compared to before (77%; p=.49)
Yeung et al. 1994	Quasi-experimental study	Non-equivalent study: two convenience samples of asthma patients on inhaling therapy	21	MDI	Intervention group given disclosure; control group not given disclosure	Adherence measured by EM using the taking adherence parameter after 2 – 3 weeks from the study start	Greater adherence in the disclosed group (81%) than in the undisclosed group (71%; p=.53)
Elixhauser et al. 1990	Randomized controlled trial	A convenience sample of psychiatric outpatients treated with lithium	90	Blister package	Experimental group received EM; control group did not	Adherence measured by self-reported, assay, % of expected prescription refills (after 2 – 4 months of study start)	Fewer expected prescription refills in the EM group (18%) than in the control group (31%; p < .01)
Cramer et al. 1990	Observational study	An unspecified sample of patients	24	MEMS	All patients received EM	Adherence measured by EM using the taking adherence parameter during the first and after a mean of 7 months from the start of the study	No difference before and after (79% vs. 79%).

### Assumption underlying valid electronic medication adherence monitoring: External validity

The fourth assumption refers to issues that might threaten the representativeness of the sample of an EM study. Examples include a large proportion of eligible subjects refusing to participate and a large proportion of patients dropping out of a study. The term dropout refers to patients who leave the study, who do not send their EM caps back to the research team, or who do not adhere to the instructions regarding EM use (resulting in unreliable EM data). Limited evidence exists concerning the external validity of EM studies in the literature. In one study, a smaller number of pill-organizer users decided to participate in EM than did non-users; the authors attributed this disparity to the burden of combining EM with pill organizers [4]. Another study found that especially patients non-adherent to the medication have difficulties also to be adherent to the guidelines of the assessment [13].

### Purpose of the study

Because no study to date has tested the four above mentioned assumptions, the aim of the present study was to examine whether these assumptions were fulfilled when using EM in a sample of kidney transplant patients. More specifically, we aimed 1) to examine the accurate functioning of EM technology, 2) to check the correspondence of recorded EM-bottle openings with the actual intake of the prescribed dose, 3) to test whether EM influenced the typical adherence behavior of patients, and (4) to examine whether using EM biased the representativeness of the sample.

## Methods

### Design, sample, and setting

Data for this prospective cohort study came from the Supporting Medication Adherence in Renal Transplantation (SMART) study, which focused on prevalence and determinants of non-adherence [22]. Patients were eligible if they had received their kidney transplant at least one year prior to enrollment, and if they were self-administering immunosuppressive medication, more than 18 years of age, German or French speaking, and literate. Patients were excluded if they were not mentally able to respond adequately to the researcher's questions or to complete the questionnaires. The convenience sample consisted of patients followed up at two outpatient transplant clinics in Switzerland. Swiss health insurance, which is compulsory, largely covers costs for immunosuppressive medications. Patients are responsible for paying out-of-pocket expenses amounting to about 10% of costs for prescribed drugs.

### Variables and measurement

We used the Medication Event Monitoring System (MEMS-5 TrackCap, Aardex Ltd., Zug, Switzerland) to measure non-adherence to immunosuppressive medications. The monitoring lasted three months and focused on one immunosuppressive drug per patient, preferably one taken twice daily (cyclosporine, tacrolimus, mycophenolate mofetil). To capture the two dosing times of patients taking a combination of azathioprine and prednisone, both of which are typically prescribed once daily, we monitored the usage of both drugs. EM bottles were prepared in the hospital pharmacy and sent to the patients. When a bottle was empty, the patient received a new one to which the EM cap could be attached. All patients received information about the monitoring of their medication-taking behavior, as requested by the ethical committee.

#### Adherence to the EM instructions

All participants received verbal and written instructions on how to use the EM system. Instructions stressed the need to match EM-bottle openings with actual drug intakes, and requested patients to describe any deviations from this guideline on the form accompanying the EM bottle. Examples of such guideline violations include inadvertently opening the EM bottle when no medication was required, stopping EM bottle use for a period, removing pills prematurely, triggering the cap while not removing medication from the bottle opening (e.g. taking medication from another source, cutting a hole in the bottom of the EM bottle and removing medication from there, ...). Upon completion of the EM measurements, we integrated the resulting patient notes into the uploaded EM data.

**Figure 2:**
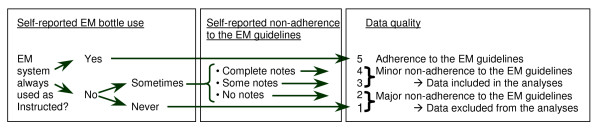
Algorithm estimating a patient's non-adherence to the EM guidelines.

At the end of the 3-month EM period, we also used a structured interview to assess perceived adherence to the EM instructions. The first goal of this interview was to detect defined periods of non-adherence to the EM instructions and to censor these data from the analysis (e.g., when a patient failed to use the EM device during the holidays for 14 days). The second goal of the interview was to assess the quality of the remaining data by scoring them according to five quality standards: 1) strict adherence to the EM guidelines (5 points); 2) self-report indicated that the EM system was not used exactly as instructed, but complete notes were available (4 points); 3) self-report indicated that the EM system was not used exactly as instructed, but incomplete notes were available (3 points); 4) self-report indicated that the EM system was not used exactly as instructed, but no notes were available (2 points); and 5) self-report indicated that neither the EM bottle nor the form was used as instructed (1 point) (Figure 2). This quality assessment tool was developed with consideration for patients' reports (recorded in field notes and later categorized) of how well they had been able to the EM use instructions. Data were considered to be of sufficient quality for analysis when the patient received a score of 3 points or higher. At the end of the monitoring period, patients were also asked whether they perceived that using EM influenced their normal medication-intake behavior, and whether this influence changed their typical adherence to the immunosuppressives positively or negatively.

#### Dropped EM caps

We asked the patients to report on their form whether they had dropped their EM caps to determine whether dropping the caps damaged the caps' recording capacity. When the patients indicated that a drop had occurred, we checked whether the recording system still functioned properly by scanning the uploaded EM data visually for extra recordings or for altered registration patterns (e.g. no further registered openings).

#### Operational definition of EM-measured adherence

Electronically measured non-adherence was evaluated for each prescribed intake moment. Two binary variables represented the taking and timing dimensions of the patients' non-adherence. The first variable indicated whether a patient omitted a dose (taking dimension). The second variable indicated whether on each expected intake moment, the monitored inter-dose interval deviated by more than 25% from the prescribed interval (timing dimension).

#### Other variables included in this study

To compare the characteristics of patients included in the EM study with those that refused to participate or dropped out, we measured non-adherence to the immunosuppressive therapy using self-report, collateral report, and blood assay. In the *self-report*, patients used a 7-point scale to score the frequency of non-adherence during the four weeks just prior to the inclusion interview – the scale ranged from never (0 points) to every day (6 points). This ordinal non-adherence variable was assessed during the inclusion interview. In the *collateral report*, nurses and physicians involved in the follow-up care of the transplant patients scored non-adherence using a 3-point scale – good adherence (1 point), fair adherence (2 points), bad adherence (3 points). We used the mean scores of the health-care workers who evaluated and scored patients. With regard to the *blood assay*, we considered one measurement moment, namely the patient's drug trough levels at inclusion in the study (i.e., of cyclosporine, mycophenolate mofetil, tacrolimus, or sirolimus before the morning dose was ingested).

### Data collection

This study was conducted in compliance with the regulations of the Ethical board of the Canton Basel-Stadt governing the protection of human subjects in research (Vereinbarung Ethikkommission beider Basel GS 300.400). The study was reviewed and approved by the ethical committee of the Canton Basel-Stadt (55/00), and the ethical committee of the Swiss Academy of Medical Sciences (PV124/00-SNF) and patients signed informed consents. We collected data from June 2001 to January 2004. Four research staff members recruited the patients, collected demographic and self-reported non-adherence data, and instructed the patients on how to use the EM system. After three months of electronic monitoring, participants received a letter to remind them to either bring back the EM device to the outpatient clinic or to send it back to the researchers (in a pre-stamped and pre-addressed envelope). Upon return of the device, we telephoned the patients and carried out a structured interview to assess their adherence to the EM guidelines (see Figure 2). During the interview, we also sought to determine how EM may have influenced the patients' typical adherence behavior. We used Powerview^® ^hard- and software to upload and adjust the EM data according to the patients' notes.

### Data analysis

We calculated the prevalence of malfunctioning EM systems (assumption 1), the prevalence of reported cap recording mismatches and non-adherence to the EM system (assumption 2), and the proportion of pill organizer users not participating (assumption 4). We also compared tabulated mean values of non-EM measured adherence between participants and non-participants/dropouts and tested for differences using the Wilcoxon-Mann-Whitney test (assumption 4).

Regarding assumption 3, we modeled the probability of non-adherence as a function of a patient's exposure time to the EM system by performing two multiple random-intercepts logistic regression analysis, one modeling dose omissions and one timing non-adherence. The random-intercepts models, which we fitted using the 'nlmixed' procedure in SAS^® ^version 9.1, accounted for the repeated measurement structure of the data. These multiple models controlled for the variables "bottle volume" (1055 cc, 325 cc, 120 cc), "the researcher who did the inclusion interview", and "the self-reported perceived EM-intervention effect" (positive or not). To get a more detailed insight into a possible non-linear course of non-adherence over time, we also fit a generalized additive model including a spline-smoothed function of exposure time (SAS 'gam' procedure) [23]. The method allowed graphical exploration of nonlinearities by leaving the relationship between non-adherence and exposure time unspecified.

## Results

**Figure 3:**
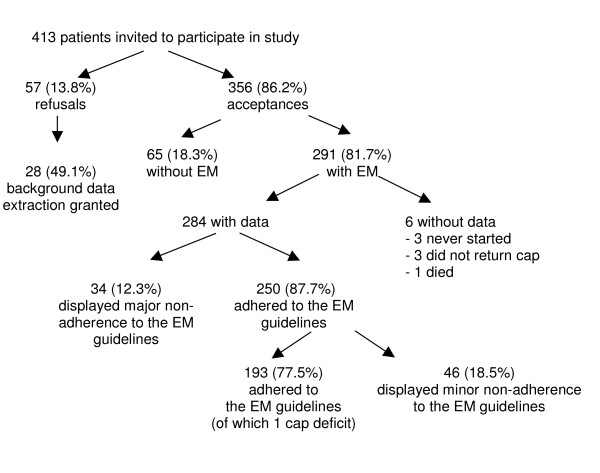
Patient-sample profile.

Four hundred thirteen adult renal transplant recipients visiting the outpatient clinic for their yearly check-up were asked to participate in the SMART study (Figure 3). Three hundred fifty-six accepted (86%) and 57 (14%) refused to participate in our study. Of the 57 patients, 28 granted us permission to obtain their demographic and clinical data from their medical files. Of the 356 participating patients, 291 (82%) agreed to be monitored electronically. The remaining 65 (18%) patients did not want to be monitored electronically but wanted to participate by completing the self-report questionnaires. Of the 291 patients who agreed to be monitored with EM, 3 (1%) never started, 3 (1%) did not return their EM caps, and one died (< 1%). Thirty-four (12%) patients were excluded from the analyses because, according to the data quality assessment outlined in the methods section and illustrated in figure 2, they failed to adhere to the EM guidelines (scores 4 and 5). The final sample consisted of 250 patients, with an average age of 54 years (sd = 13). The majority of subjects were Swiss citizens (n = 209; 84%) and male (n = 142; 57%). Immunosuppressive therapies consisted of cyclosporine and mycophenolate mofetil (n = 71; 28%), cyclosporine (n = 38; 15%), cyclosporine and azathioprine (n = 37; 15%), or other combinations (n = 104; 42%). Further details regarding the sample characteristics can be found in Table 2.

**Table 2:** Characteristics of the sample (n = 250)

**Variable**	**Categories**	**Value**
Age		Mean= 54 (sd = 13)
Gender	Male	142 (56.8%)
Living alone	No	194 (77.6%)
Employed	Yes	130 (52.0%)
Education	until age 11/12 years	33 (13.2%)
	until age 12/13–14/15 years	118 (47.2%)
	until age 15/16–18/19 years	26 (10.4%)
	advanced (college)	73 (29.2%)
Nationality	Swiss	209 (83.6%)
Immunosuppression	Cyclosporine & mycophenolate mofetil	71 (28.4%)
	Cyclosporine	38 (15.2%)
	Cyclosporine & azathioprine	37 (14.8%)
	Azathioprine & prednisone	18 (7.2%)
	Azathioprine & tacrolimus	14 (5.6%)
	Other combinations	72 (28.8%)
Monitored immunosuppressives	Mycophenolate mofetil	103 (41.2%)
	Cyclosporine	89 (35.6%)
	Azathioprine/prednisone	19 (7.6%)
	Tacrolimus	37 (14.8%)
	Sirolimus	2 (0.8%)
Self-reported EM influence on typical adherence	No influence	188 (76.1%)
	Positive influence	53 (21.5%)
	Negative influence	6 (2.4%)

### Assumption 1

Sixty-one (24%) patients reported that they had dropped their EM caps. None of these caps registered the drop as an event, nor did the data afterwards reflect any visually detectable signs of damage to the recording system. One patient (0.4%) who never dropped his cap claimed to have better adherence than suggested by his EM data. We manually checked his cap, which revealed that it failed to register openings. The manual check was done by one of the investigators, who unscrewed the cap from the bottle for one week and downloaded the registrations onto the computer. No openings were registered. This cap seemed to have gradually lost its registration capacity during the 3-month monitoring period, because all expected openings within the first two weeks of the measurement period were recorded, after which the event recordings declined. Although the gradual decline of cap function suggested a battery problem, a battery check did not reveal an exhausted battery.

### Assumption 2

Of the 249 patients with reliable EM data (= 250 minus the failed cap), 155 (62%) reported discrepancies on their form between recorded openings and actual medication intakes, which required 1084 adjustments to the 44761 events of the final data base (2.4 adjustments, on average, per person). Twenty-eight percent of the adjustments involved early decants of pills that were ingested later. The most frequently mentioned reasons patients gave for the discrepancies were going out, being on a trip, and having a meeting. Other reasons for correspondence failures were taking medication from another supply, phantom openings to demonstrate the EM bottle to visitors, and opening the wrong bottle. Twenty-three patients (9.2%) had defined periods of non-adherence to the guidelines of correct EM use according to the used data quality algorithm (figure 2). For these patients, an average of 13.6 days was censored from the total monitoring period. Censoring these days increased the average percentage of correctly dosed days from 92.9% to 96.3%.

### Assumption 3

**Figure 4:**
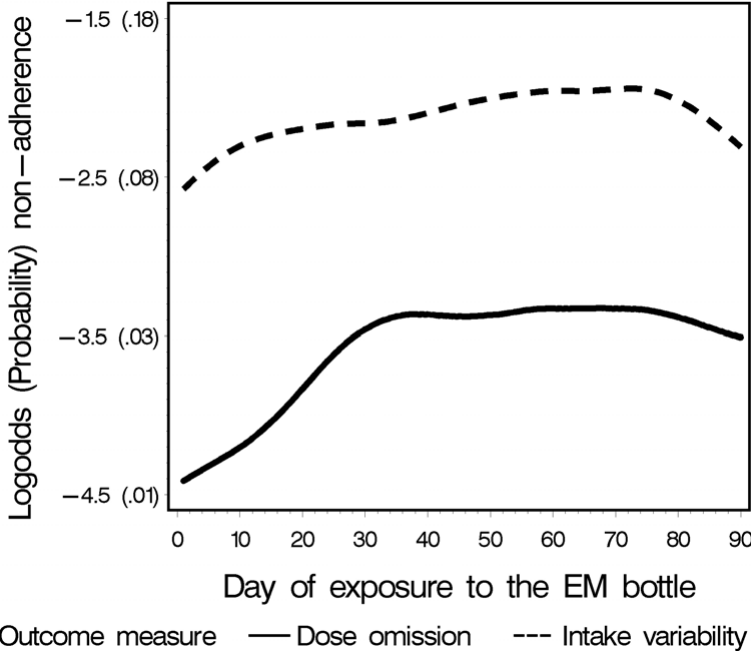
Observed course of non-adherence over time.

The random-intercepts logistic regression analysis confirmed an increase in both taking and timing non-adherence over time (Table 3). The odds on non-adherence increased over one month by about 30% for taking (OR: 1.31; 95%CI: 1.17–1.46) and 25% for timing adherence (OR: 1.26; 95% CI: 1.17–1.35). In addition, the nonlinear regression lines showed that the increase in both dimensions mainly occurred during the first 5 weeks of monitoring (Figure 4). After day 35, the taking dimension of non-adherence stabilized. The average percentage of correctly dosed days was 96.7% when including the entire 3-month measurement period, but slightly decreased to 96.3% when the first 35 days were excluded. The timing dimension of non-adherence stabilized after about day 50. The average percentage of correctly timed intakes was 91.8% when including all data points, and slightly decreased to 91.4% when only considering the stable phase between day 50 and 75. A post hoc analysis identifying potential interactions between exposure to EM and perception of the EM-intervention effect, showed a stronger EM-intervention effect in patients acknowledging an intervention effect than in patients stating that they experienced no intervention effect (p = 0.003).

**Table 3:** Estimates and inferences from the multiple logistic random-intercept models predicting the chance of non-adherence

**Outcome variable**	**Parameter**	**Estimate**	**Standard error**	**Odds ratio (95% confidence interval)**	**DF**	**t value**	**p value**
Omitted intakes	Random-intercepts variance	2.845	0.445		241	6.39	< .0001
	Intercept	-5.900	0.470		241	-12.54	< .0001
	Exposure to EM (per month)	0.270	0.055	1.31 (1.17–1.46)	241	4.91	< .0001
	Bottle size (per 100 ml)	0.014	0.040	1.01 (0.94–1.10)	241	0.93	0.35
	Influence perception	0.277	0.328	1.32 (0.69–2.52)	241	0.84	0.39
	Interviewer 1 vs. interviewer 4	0.461	0.356	1.59 (0.79–3.19)	241	1.29	0.19
	Interviewer 2 vs. interviewer 4	0.011	0.365	1.01 (0.50–2.07)	241	0.03	0.97
	Interviewer 3 vs. interviewer 4	0.022	0.438	1.02 (0.43–2.42)	241	0.05	0.95
Intake variability	Random-intercepts variance	3.486	0.422		241	8.26	< .0001
	Intercept	-3.033	0.414		241	-7.31	< .0001
	Exposure to EM (per month)	0.227	0.036	1.26 (1.17–1.35)	241	6.67	< .0001
	Bottle size (per 100 ml)	-0.106	0.037	0.90 (0.84–0.97)	241	-2.08	0.04
	Influence perception	0.011	0.314	1.01 (0.54–1.88)	241	0.04	0.97
	Interviewer 1 vs. interviewer 4	0.704	0.340	2.02 (1.04–3.95)	241	2.07	0.04
	Interviewer 2 vs. interviewer 4	0.008	0.344	1.01 (0.51–1.99)	241	0.02	0.98
	Interviewer 3 vs. interviewer 4	0.148	0.422	1.16 (0.50–2.67)	241	0.35	0.73

### Assumption 4

A comparison of pill organizer use in participants vs. non-participants in the EM part of our study showed that pillbox use was more common among non-participants (38.5%) than among participants (25.1%: p = 0.03). Table 4 compares non-EM adherence measurements of participants and non-participants/dropouts (i.e., self-report, collateral report and blood assay). We did not find any statistically significant evidence that dropouts or non-participants had a lower adherence to the immunosuppressive therapy than participants.

**Table 4:** Adherence comparison between participants/non-participants and participants with reliable EM data and participant dropouts

	Variable	Subgroups					
Non-participants		Non-participants			Participants		
		
		median	iqr ^b^	n	median	iqr	n
		
	Self-report ^a^	0.0	0.0	65	0.0	0.0	284
	Collateral report ^a^	1.0	0.1	35	1.0	0.3	164
	Assay: cyclosporine (mmol/l)	112.5	50.0	50	105.0	56.0	191
	Assay: tacrolimus (mmol/l)	7.6	3.2	21	7.2	3.9	44
	Assay: sirolimus (mmol/l)	14.3	4.2	5	8.8	9.5	15
	Assay: mycophenolate mofetil (mmol/l)	3.3	1.8	41	2.6	1.9	122

Dropouts		Non-adherers to the EM guidelines			Adherers to the EM-guidelines		
		
		median	iqr	n	median	iqr	n
		
	Self-report	0.0	0.0	36	0.0	0.0	244
	Collateral report	1.0	0.7	35	1.0	0.3	224
	Assay: cyclosporine (mmol/l)	108.0	43.0	25	104.5	58.5	164
	Assay: tacrolimus (mmol/l)	6.2	6.1	5	7.2	3.7	39
	Assay: mycophenolate mofetil (mmol/l)	2.7	1.8	17	2.6	1.9	101

^a ^Definition: see section variables and measurement

^b ^Interquartile range

## Discussion

This study examined four assumptions underlying the valid electronic measurement of medication non-adherence, which, if violated, might threaten the external and/or internal validity of EM studies.

### Assumption 1

We identified one EM device that had stopped recording cap openings during the study (0.4%). The failure rate is similar to that reported on the Aardex website (< 0.5%), and confirms literature reports that EM devices used in studies can be damaged [6,7]. Although our study evaluates the MEMS-5 monitors, and not the newer MEMS-6 monitors Aardex refers to, MEMS-6 differs from MEMS-5 mainly in its data upload technology. MEMS-6 is comparable to its predecessor regarding most other features [24]. The result of our study can thus be considered representative for the system currently on the market. The existence of a non-registering cap shows that non-adherence overestimation is possible for a small number of patients who have damaged caps that remain undetected. A systematic check of the recording system before and after a monitoring period is advisable.

### Assumption 2

The present study revealed that EM registrations in our sample often did not correspond to the actual ingestion of the monitored medication. Our results are similar to a study of HIV patients, where 36% failed to use the EM bottles continuously during a one-year monitoring period, 38% used their EM bottles only on occasion, and 3% always removed more than one dose per EM-bottle opening [6]. As already suggested previously in a study using fabricated data [8], we showed that choosing to censor time periods clearly affects the obtained prevalence of non-adherence. Our uncensored data showed a 3.4% higher non-adherence than the censored data, which could be considered as an overestimation of non-adherence. Due to technical reasons, we were not able to also examine the effect of data adjustment (based on the forms accompanying the EM bottle), but it may be safely assumed that the non-adherence overestimation would have been even larger if we also omitted the patient-reported discrepancies. Nevertheless, a study supported by Aardex showed that uncorrected EM monitoring enables accurate estimation of patient's drug exposure. How accurately patients adhered to the EM instructions is not known [25]. The fact that in our study, patients seem to not always adhere fully to the guidelines for MEMS use is not necessarily a drawback of the idea of EM itself. However, one can conceive of a system that is easier to use and invokes less resistance [6]. A more practical, easy-to-use system may increase EM use outside of the home, thereby increasing the correspondence between event registration and pill ingestion. Recent efforts are being made in this regard in that several companies have started to market EM-blister packs that can be easily carried along: Bang & Olufsen's IDAS^®^, IMC's Med-ic^®^, and MeadWestvaco's Cerepak^®^.

We are aware that our attempts to estimate the adherence of patients to the EM instructions rely on patient self-reports, which is not a highly sensitive measurement method. However, in the case of the patient reports we used to supplement missing or correct phantom registrations, recall bias could not have been a large problem because the notes were recorded at the time of each event or not long thereafter. The interview at the end of the measurement period asked for a general impression of the use of the bottle and the notes form, not for detailed adherence information. Even if less reliable than other sources, this kind of patient report may result in a more accurate measurement than simply not correcting the data with information given by the patient. When a patient for instance assures that during a holiday period the monitor was left at home while medication was taken from another source, it would be less accurate to discard this information and rely blindly on the monitor's records than to use this information to interpret the missing data in the EM records. Future studies should be clear about how they dealt with issues of non-adherence to the EM system. Few studies to date report on these issues. Because the decisions made by the investigators to clean their data have an effect on the obtained results, and may even introduce other forms of bias, more openness is needed in future research. Investigators may be helped in this regard by a recently published checklist that aims at conceptually clarifying EM data management decisions [8].

### Assumption 3

Testing the third assumption revealed that EM might have influenced adherence behavior. The prevalence of non-adherence was very low in the beginning of the monitoring period. A subsequent increase of non-adherence probably reflects the waning of the adherence-enhancing effect of introducing EM to patients' daily lives, although our sensitivity analysis showed that omitting the first month did not lead to a large increase of the prevalence of non-adherence, implying that the observed intervention effect had only minor clinical relevance. The absence of a control group prevents drawing firm conclusions, but the fact that our research group found the same pattern in HIV patients strengthens this hypothesis [26]. Traditional analysis approaches, using period prevalence parameters like "the percentage of prescribed medications that are taken", are limited with regard to analysis of detailed time-dependent evolutions. Moreover, their often J-shaped distributions force researchers to rely on simple nonparametric tests [27]. Although we only found minimal differences in prevalences including the intervention period compared to prevalences excluding it, bias may increase when the proportion of the intervention period is high compared to the whole monitoring period, which may be the case with studies lasting only a month. Studies assessing patients' normal level of adherence should therefore examine presence and duration of an intervention effect using longitudinal analysis techniques [28]. It has to be noted that all ethically permissible adherence assessment methods, electronic or otherwise, require the consent of the subject, and therefore influence the observed behavior. It is well-documented, for example, that blood assays lead to white-coat adherence [29], and that self-report also influences the self-reportet adherence behavior [30]. The fact that our researchers were not blinded may also have influenced the occurrence and strength of the intervention effect. The found significant difference in patient's timing adherence between two of the researchers (Table 3), may be an indication of this influence. Differences could occur if patient recruitment was accompanied by stressing on the importance of being adherent to the immunosuppressive regimen.

### Assumption 4

We found little evidence of compromised *external validity *in this sample of renal transplant patients. Our study confirmed previous research showing less agreement to participate in EM assessment where patients said they used a pill organizer, suggesting selection bias [4]. This may be because subjects perceive an extra burden in managing an organizer and a MEMS bottle at the same time. Our study failed to confirm the hypothesis that patients who did not adhere to EM guidelines, display higher medication non-adherence than patients who did adhere to the guidelines [13]. This does not necessarily mean that there was no difference: measurement error coming from small sample sizes, low sensitivity of non-EM non-adherence assessment [1], and low sensitivity of our EM-data quality assessment, may have blurred existing differences. This assumption should be further investigated.

## Conclusion

This article on the validity of electronic monitoring of non-adherence to medication shows the challenge of disentangling non-adherence measurement from processes that bias the EM measurement. We hope that our study helps to increase awareness among adherence researchers of the complexity of electronically monitoring medication taking, and that it acts as an impetus for the improvement of EM use. We offered a set of requirements essential to enhance methodological quality of future EM studies. Specifically, EM studies should 1) perform a systematic functionality control of the EM system before and after use; 2) assess adherence to the EM guidelines; 3) examine intervention effects; and 4) examine sample representativeness. Meeting these methodological standards plus being transparent in the reporting on the exact operationalization of EM, may help EM to approach its gold-standard aspirations. Although the population used in this study is not representative of all populations in which MEMS is used, the approach we present could be used as a model for testing assumptions in other populations.

## List of abbreviations

EM: Electronic Monitoring

HIV: Human Immunodeficiency Virus

MDI: Metered-Dose Inhaler

MEMS: Medication Event Monitoring System

RCT: Randomized Controlled Trial

SMART: Supporting Medication Adherence in Renal Transplantation

## Competing interests

The author(s) declare that they have no competing interests.

## Authors' contributions

SDG and JS were the principal investigators of this study.

KD drafted the manuscript and carried out the analyses.

PSK and SDG developed the algorithm to measure adherence to the EM guidelines.

JY participated in the data analysis and the interpretation of the results.

AB, JS, KD and SDG participated in the conception and design of the study, and commented on earlier versions of the manuscript.

All authors read and approved the final manuscript.

## Pre-publication history

The pre-publication history for this paper can be accessed here:


